# General practitioners’ and out-of-hours doctors’ role as gatekeeper in emergency admissions to somatic hospitals in Norway: registry-based observational study

**DOI:** 10.1186/s12913-019-4419-0

**Published:** 2019-08-14

**Authors:** Jesper Blinkenberg, Sahar Pahlavanyali, Øystein Hetlevik, Hogne Sandvik, Steinar Hunskaar

**Affiliations:** 1 National Centre for Emergency Primary Health Care, NORCE Norwegian Research Centre, Kalfarveien 31, 5018 Bergen, Norway; 2 Department of Global Public Health and Primary Care, University of Bergen, Kalfarveien 31, 5018 Bergen, Norway

**Keywords:** Norway, General practitioners, After-hours care, Out-of-hours medical care, Gatekeeping, Referral and consultation, Emergencies, Patient admission

## Abstract

**Background:**

Primary care doctors have a gatekeeper function in many healthcare systems, and strategies to reduce emergency hospital admissions often focus on general practitioners’ (GPs’) and out-of-hours (OOH) doctors’ role. The aim of the present study was to investigate these doctors’ role in emergency admissions to somatic hospitals in the Norwegian public healthcare system, where GPs and OOH doctors have a distinct gatekeeper function.

**Methods:**

A cross-sectional analysis was performed by linking data from the Norwegian Patient Registry (NPR) and the physicians’ claims database. The referring doctor was defined as the physician who had sent a claim for a consultation with the patient within 24 h prior to an emergency admission. If there was no claim registered prior to hospital arrival, the admission was defined as direct, representing admissions from ambulance services, referrals from nursing home doctors, and admissions initiated by in-hospital doctors.

**Results:**

In 2014 there were 497,587 emergency admissions to somatic hospitals in Norway after excluding birth related conditions. Direct admissions were most frequent (43%), 31% were referred by OOH doctors, 25% were referred by GPs, whereas only 2% were referred from outpatient clinics or private specialists with public contract. Direct admissions were more common in central areas (52%), here GPs’ referrals constituted only 16%. The prehospital paths varied with the hospital discharge diagnosis. For anaemias, 46–49% were referred by GPs, for acute appendicitis and mental/alcohol related disorders 52 and 49% were referred by OOH doctors, respectively. For both malignant neoplasms and cardiac arrest 63% were direct admissions.

**Conclusions:**

GPs or OOH doctors referred many emergencies to somatic hospitals, and for some clinical conditions GPs’ and OOH doctors’ gatekeeping role was substantial. However, a significant proportion of the emergency admissions was direct, and this reduces the impact of the GPs’ and OOH doctors’ gatekeeper roles, even in a strict gatekeeping system.

## Background

An aging population and new diagnostic and therapeutic possibilities, combined with growing expectations, put extra demands on the healthcare system. Emergency hospital admissions represent a considerable workload and expense for the healthcare systems worldwide. Reducing these admissions has been a priority for many years [1–6]. Several studies have described various factors influencing the rate of emergency admissions, and a variety of factors has been found to be associated with excess of admissions or avoidable admissions [7–9]. Age older than 65 years is associated with higher emergency hospital admission rates in the UK and US [7, 10, 11]. On the other hand, continuity of care in general practice and access to a preferred general practitioner (GP) have been shown to reduce the emergency admission rates in general [4, 7, 9], and also for ambulatory care sensitive conditions [12]. There is variation in admission rates by clinical condition in the US [13]. However, analyses of the overall picture of prehospital paths and effects of gatekeeping have received less attention.

GPs are gatekeepers in many healthcare systems. Gatekeeping means that patients have to see a primary care provider who decides whether specialist care is necessary. Such referral regulates the access to specialty care, hospital care, or diagnostic tests. It is supposed to give better control over the healthcare costs and more targeted and efficient hospital healthcare [14]. It has been found to lower utilization of healthcare services and expenditures [15].

Access to specialist healthcare in Norway is generally referral based, and patients cannot meet at hospital emergency rooms in Norway without a prior contact with prehospital healthcare [16]. This makes the Norwegian healthcare system well suited to study the impact of strict gatekeeping on emergency admissions. A Norwegian study from a single hospital indicated that patients admitted for emergencies to a medical department often did not have any contact with GPs or out-of-hours (OOH) doctors prior to the admission [17]. However, a nationwide analysis of the prehospital paths for emergency hospital admissions in a public healthcare system where GPs and OOH doctors have a distinct gatekeeper function, like Norway, has not been conducted.

The aim of the present study was to investigate the prehospital paths for emergency admissions to somatic hospitals in Norway and describe variations in the gatekeeping role of the GPs and OOH doctors with respect to geographical centrality and time of day. In addition, we wanted to explore GPs’ and OOH doctors’ role in emergency admissions to hospital in relation to the clinical conditions involved.

## Methods

The study was designed as a registry based cross-sectional analysis using data from the total population in Norway.

### Norwegian healthcare system

All Norwegian residents have access to a public healthcare system, covered by the National Insurance Scheme. Patients older than 15 years have to pay an out of pocket fee for consultations with GPs, OOH doctors, ambulatory care specialists, and outpatient clinics in hospitals (15–33€ in 2014). There is a maximum sum (219 € in 2014) on how much a patient may have to pay during one calendar year [16]. Hospital stays and ambulance services are free of charge.

The municipalities organize the primary healthcare, including GPs and OOH services, while the state is in charge of hospitals and the ambulance services [16, 18]. In 2001, the Norwegian government established a patient list scheme with Regular General Practitioners (RGP scheme). The Norwegian Health Economics Administration (HELFO) is administrator for the scheme, which provides a personal RGP for every resident [19].

RGPs provide medical care for their patients during office hours, both in acute and non-acute cases [19, 20]. OOH services provide healthcare in case of emergencies 24 h a day by consultations, home visits and callouts, also when the RGPs’ practices are closed [21]. In 2014, there were 191 OOH services in Norway, 80 were organized as municipal operations and 111 as inter-municipal cooperation [22]. The RGPs are obliged to participate in the OOH services [20]. In addition, some interns and doctors with other specialties also work at OOH services.

If a life-threatening condition is suspected, the public can call 113 – the emergency medical communication centre (EMCC). In case of less serious conditions, GPs can be contacted during office hours, and OOH services are accessible at all times at the national number 116117. The EMCC and OOH services work closely connected through a national emergency radio network. Depending on the symptoms’ presentation, the EMCC decides whether the patient needs ambulance transport directly to hospital, or should be seen by another healthcare provider, like a GP or OOH doctor. The OOH service usually has a call-first routine, but at some places, patients may show up directly.

### Study setting

Based on data from all registered inhabitants during 2014 in Norway (*N* = 5,109,056) we identified all emergency admissions to Norwegian hospitals in the period from 1 January until 31 December 2014. As psychiatric hospitals were not included in the study, we use the term somatic hospital admissions. Three national registries were used as data sources; Statistics Norway (SSB), Control and Payment of Reimbursement to Health Service Providers database (KUHR), and The Norwegian Patient Registry (NPR).

SSB contains official demographic data about the Norwegian population. SSB has classified all municipalities based on centrality, which is a description of a municipality’s geographical position in relation to workplaces and public services. The classification gives every municipality a value from 0 to 1000. Based on this value the municipalities are then categorized into 6 groups, with group 1 representing the most urban municipalities in the capital region, and group 6 referring to the most rural municipalities [23].

The KUHR database is administrated by HELFO, which receives compensation claims from all GPs, OOH doctors, and private specialists with public contract (PSPC). These claims are registered together with additional information about care provider’s ID-number, patient’s ID-number, diagnosis, gender, age, address, date and time and type of service provided (consultation, home visit or telephone consultation). GP contacts and OOH contacts are coded separately.

NPR records information about all the patients’ contacts with specialist healthcare, including information about the patient’s ID number, gender, age, date and time and type of service performed, including institution, degree of urgency, and discharge diagnosis. For some administrative reasons, NPR also included information from the OOH services in the second largest city (Bergen), and these contacts were in this study included as OOH service contacts.

Contacts with other medical services, such as nursing home doctors, private medical providers, or the ambulance services, are not included in these registries.

SSB pseudo anonymized the 2014 population data by replacing the patient’s ID-number with a serial number. This number was then sent to NPR and HELFO, and these registries also replaced the ID-number with the same serial number. Thus, data from all three sources could be combined.

### Variables and definitions

NPR categorizes every admission according to degree of urgency. We defined an emergency admission as a patient requiring hospital admission immediately or within 24 h after the contact determining admission is necessary.

NPR contains no variable for referring agent. Therefore, we made a proxy for this by linking each admission to a prehospital contact if the contact was within 24 h prior to the time of admission. In case of admission on a Monday, a contact during the preceding weekend was accepted as the referral contact. Since GPs and OOH doctors are not always able to fill out the claims when seeing the patient in emergency consultations, delayed compensation claims produced within 12 h after the admission time was also defined as a referral contact.

For some admissions, there were more than one contact prior to the admission. These contacts were prioritized and included in the following order: OOH contact, GP contact, outpatient contact, and PSPC contact, reflecting that an OOH contact may be assumed to be the most urgent contact.

The emergency admissions were then categorized into four prehospital paths, according to the healthcare services that had provided the gatekeeping or the referral service. The admission was recognized as (1) a GP admission, (2) an OOH doctor admission, or (3) a PSPC admission, if the patient had seen one of these services a short time before admission, respectively. If there was no such contact found prior to the admission, it was categorized as (4) a direct admission.

Weekday was defined as Monday to Friday, and weekend as Saturday and Sunday, corresponding to GPs opening hours. Public holidays were also defined as weekend.

The prehospital paths were analysed based on the International Statistical Classification of Diseases and Related Health Problems version 10 (ICD-10) [24]. The admissions were presented by diagnosis chapters using the first letter in the ICD-10 codes. When analysing more specific diagnoses we used the first three characters of the diagnosis code, thus reducing the number of diagnoses.

When analysing discharge diagnoses typical for GP contacts or OOH contacts prior to admission or diagnoses for direct admissions, we excluded diagnoses with less than 500 cases. Some diagnoses (ICD-chapters) were expected to be the result of direct hospital follow-ups, and were excluded: O (pregnancy, childbirth, and the puerperium) and Z (persons encountering health services for examination and investigation). Chapter C (malignant neoplasms) showed a specific pattern and was therefore analyzed as one unit.

**Fig. 1:**
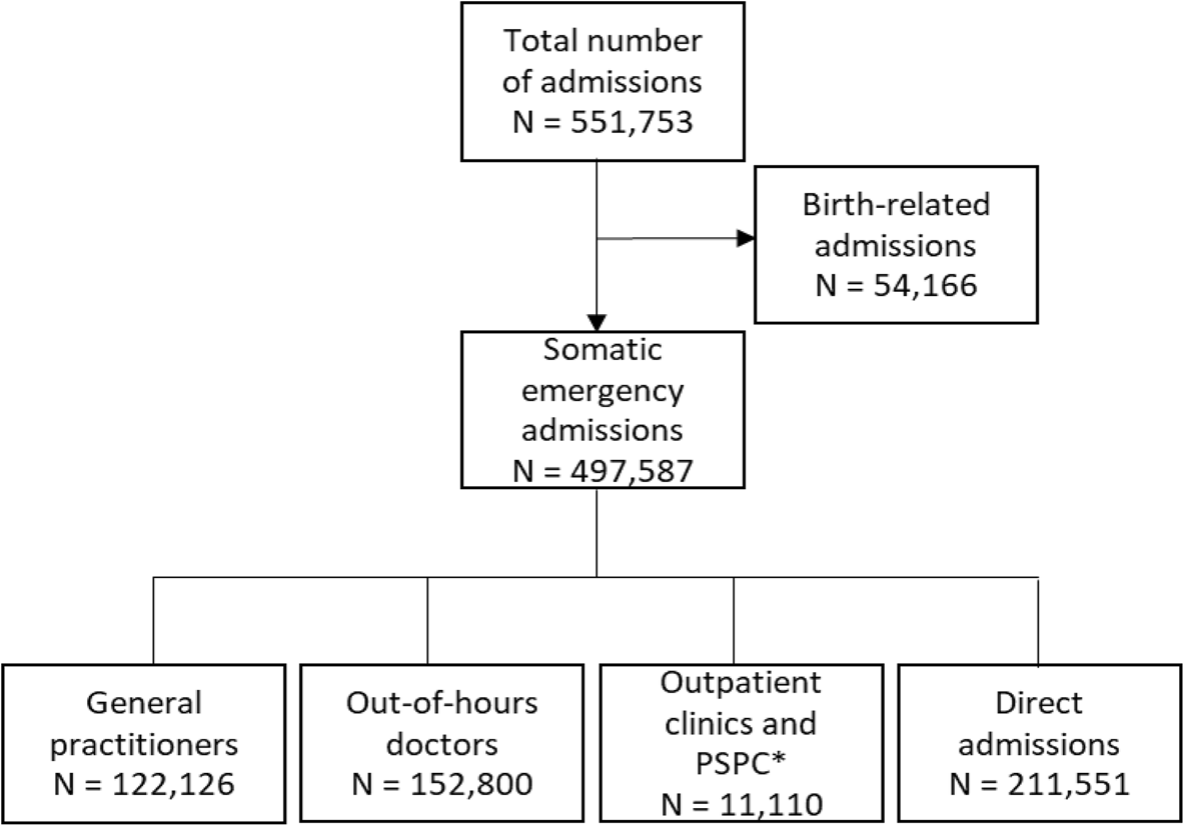
Prehospital pathways for emergency admissions. Legend: Prehospital pathways for all the emergency admissions to somatic hospitals in Norway in 2014 *Private specialist with public contract

According to national routines on maternity care, women in labour can contact hospital directly for admission to a maternity ward. A birth-related admission was defined as either an admission with the primary discharge ICD-10 diagnosis “Outcome of delivery” (Z37) or “Liveborn infant according to place of birth and type of delivery” (Z38). All admissions in the diagnosis chapter containing conditions originating in the perinatal period (P) were also defined as a birth-related admission. The large majority of birth admissions were identified as direct admissions and were excluded from further analyses (Fig. 1). However, birth related admissions with a GP or OOH contact prior to admission, were kept as a GP or OOH contact.

### Analyses

The analyses were carried out by using Stata® 15.0 (Stata Corp., College Station, TX, USA). A flow chart was constructed for the predefined prehospital paths. Prehospital paths, discharge diagnoses, and centrality were analysed by frequency two-way tables. As the material is a complete national data set, all differences are real and without statistical uncertainty. The results are therefore presented without any statistical tests.

## Results

There were 551,753 emergency hospital admissions to somatic hospitals in Norway in 2014, according to our case definition. One in ten admissions were birth related, hence not supposed to have visited a primary healthcare doctor before admission (Fig. 1). After excluding the birth-related admissions from the material, the distribution of the remaining 497,587 somatic emergency hospital admissions by referring agents is shown in Fig. 1. Direct admissions were most frequent (43%), 31% were referred by OOH doctors, 25% were referred by GPs, whereas only 2% were referred from outpatient’s clinics or PSPCs.

### Day and time of admission

**Fig. 2:**
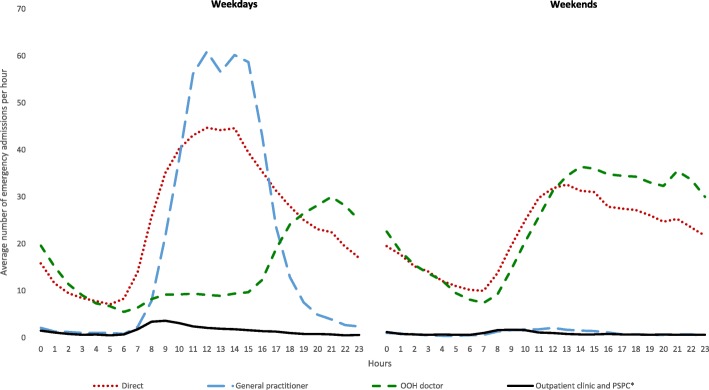
Emergency admissions by prehospital pathway and time of the day. Legend: All emergency admissions to somatic hospitals in 2014 in Norway, sorted by prehospital pathways and time of the day during weekdays and weekends

Large differences in prehospital paths were found for weekdays vs. weekends, and by day and night hours (Fig. 2). On weekdays, most patients were admitted during the daytime, 59% from 8 am to 4 pm. GP contacts were the main prehospital path in this period, with a little dip representing lunch hour. No patients were admitted from GPs during weekends. Patients referred from the OOH services were the largest group during evenings and nights on weekdays, and from midday until 2 am during afternoons and nights on weekends. Direct admissions were dominating during morning hours, both weekdays and weekends.

### Centrality patterns

Tables 1 and 2 show emergency admissions by centrality group, referring agent, and per 1000 inhabitants. The mean number of emergency admissions per 1000 inhabitants per year was 97, highest in the least central group (115), and lowest in the most central group (87). For direct admissions, we found an increasing proportion by increasing centrality, so in the most central (urban) areas more than half of the admissions to somatic hospitals in 2014 were direct admissions. For the two least central areas, with 12% of the population and 14% of the admissions, only 37% of the admissions were direct.

**Table 1:** Frequency of all emergency admissions to somatic hospitals in Norway 2014 by patient residence centrality

	All admissions		Population	
Centrality	N	%	N	Admissions per 1000
1 (most central)	88,050	18	1,011,602	87
2	121,976	25	1,199,290	102
3	123,990	25	1,357,164	91
4	94,407	19	906,580	104
5	48,956	10	459,368	107
6 (least central)	20,092	4	175,052	115
Sum	497,471^a^	100	5,109,056	97

^a^ 116 cases missing the centrality variable

**Table 2:** Variation in prehospital paths by patient residence centrality for all emergency admissions to somatic hospitals in Norway 2014 (*N* = 497,587^a^)

	General practitioner		Out-of-hours doctor		Outpatient clinic or PSPC^b^		Direct admission	
Centrality	N	%	N	%	N	%	N	%
1 (most central)	13,838	16	24,804	28	4038	5	45,370	52
2	28,695	24	39,335	32	2271	2	51,675	42
3	32,060	26	37,024	30	2241	2	52,665	42
4	26,397	28	29,909	32	1675	2	36,426	39
5	14,972	31	15,458	32	667	1	17,859	36
6 (least central)	6156	31	6226	31	217	1	7493	37

^a^ 116 cases missing the centrality variable

^b^ Private specialist with public contract

There was an increasing proportion of referrals from GPs by decreasing centrality, as referrals from GPs constituted only 16% in the most central group and 31% of the admissions in the two least central groups of municipalities. The proportion of patients referred from OOH doctors was relatively stable by centrality group, varying from 28 to 32% in the various centrality groups. Outpatient clinics and PSPCs referred few patients, and had low shares in all centrality groups, but reached 5% in the most central group. Hospitals in the most central regions had up to 61% direct admissions, whereas the most rural had only 29% (data not shown).

### Diagnoses

**Fig. 3:**
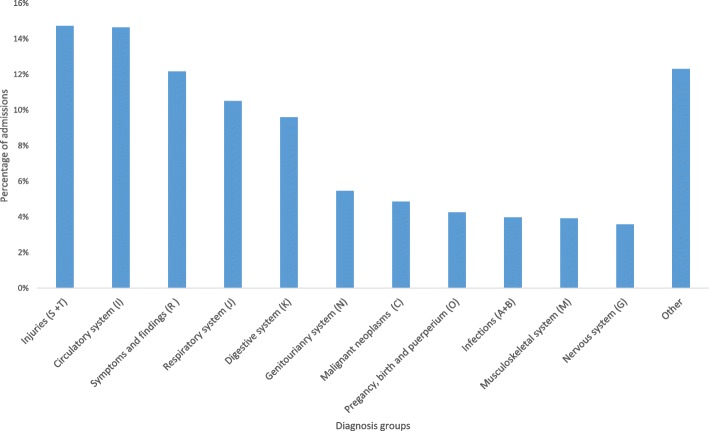
Emergency admissions by diagnosis groups. Legend: Distribution of admissions by diagnosis groups for the discharge diagnosis (ICD-10) after emergency admissions to somatic hospitals (except normal birth and related conditions) in Norway 2014 (*N* = 497,587)

Among all the emergency admissions, injuries were the most frequent discharge diagnosis group, followed by diseases in the circulatory system, symptoms and findings not elsewhere classified, and diseases in the respiratory system (Fig. 3).

Table 3 shows the 20 most common diagnoses by the four prehospital paths, these diagnoses constituted 35% of all admissions. Pneumonia (J15, J18) was the most common diagnosis, followed by pain in throat and chest (R07), abdominal and pelvic pain (R10), atrial arrhythmias (I48), and acute myocardial infarction (I21). Several kinds of injuries were also in the top 20, together with major chronic diseases such as chronic obstructive pulmonary disease (COPD) and heart failure.

**Table 3:** Distribution of prehospital pathways for all admissions (except birth related conditions), and by discharge diagnosis (ICD-10 codes) for the 20 most common diagnosis after somatic hospital stays in Norway 2014

	General practitioner		Out-of-hours doctor		Outpatient clinic or PSPC^a^		Direct admission		Sum	
	N	%	N	%	N	%	N	%	N	%
All admissions	122,126	25	152,800	31	11,110	2	211,551	43	497,587	100
Diagnosis (ICD-10)										
Pneumonia (J15 + J18)	5595	27	6557	32	3161	1	8198	40	20,488	100
Pain in throat and chest (R07)	4332	27	6613	41	138	1	5287	32	16,320	100
Abdominal and pelvic pain (R10)	4538	29	7163	46	88	1	3723	24	15,518	100
Atrial fibrillation and flutter (I48)	3990	34	3314	28	94	1	4391	37	11,873	100
Acute myocardial infarction (I21)	2386	21	3115	28	178	1	5694	50	11,310	100
Fracture of femur (S72)	1240	12	2684	27	115	2	5821	58	9958	100
Chronic obstructive pumonary disease (J44)	2350	26	2897	32	213	1	3705	41	9003	100
Intracranial injury (S06)	1045	13	3276	40	51	4	3595	44	8249	100
Other dissorders of urinary system (N39)	1899	25	2697	36	333	1	2842	38	7498	100
Cerebral infarction (I63)	1687	23	1835	25	60	1	3831	52	7409	100
Heart failure (I50)	2579	35	1874	25	56	1	2859	39	7392	100
Angina pectoris (I20)	1915	28	1922	28	80	2	2794	41	6750	100
Complications of procedures (T81)	1139	20	1338	23	119	3	3151	54	5820	100
Alcohol related disorders (F10)	546	9	2838	49	192	0	2368	41	5779	100
Acute appendicitis (K35)	1686	30	2958	52	27	0	987	17	5642	100
Syncope and collapse (R55)	1177	22	1954	37	11	1	2108	40	5294	100
Choleolithiasis (K80)	1424	28	2193	44	55	1	1355	27	5002	100
Medical observation (Z03)	1383	28	1527	31	30	1	1945	40	4914	100
Fracture of forearm (S52)	629	13	1799	38	59	7	2013	42	4777	100
Fracture of lower leg, including ancle (S82)	562	12	1645	35	228	5	2247	48	4682	100
Sum	42,102		60,199		2463		68,914		173,678	35 (of all)

^a^ Private specialist with public contract

Prehospital paths differed considerably between different discharge diagnoses (Table 4). The GPs (25% of all emergency admissions) had a much higher share of, e.g. anaemias and other conditions of the blood, sciatica, heart failure, and various local subacute diseases like haemorrhoids, diverticulitis, and deep venous thrombosis. OOH doctors (31% of all admissions) had a high share of referrals for various acute conditions, like appendicitis, foreign body in alimentary tract, mental and alcohol related disorders, abdominal pain and other acute gastro-intestinal conditions, asthma, and nephrolithiasis. The direct prehospital path (43% of all admissions) was most common for the diagnosis of agranulocytosis, hydrocephalus and cardiac arrest, but all with relatively small absolute numbers. All diagnoses on the top 20 list for direct admissions had a percentage above 50, revealing a list of conditions being extensively removed from undergoing a gatekeeper process. Admissions for malignant neoplasms was by far the largest group(C) (63%, *N* = 24,190), followed by fractures and other orthopedic conditions, epilepsy, and chronic diseases of the lungs, kidneys and heart. Major and common emergencies, such as stroke (52%), acute myocardial infarction (50%) and pneumonia (40%) did not reach the top 20 list of direct admissions but had high absolute numbers.

**Table 4:** Emergency admissions by discharge ICD-10 diagnosis where contact with a) GP or b) out-of-hour (OOH) doctor, or c) direct admission is the dominating prehospital pathway

a) GP contact before admission (*N* = 122,126)		
	Admissions with the discharge diagnose	GP contact before admission
Diagnosis	N	%
Iron deficiency anaemia (D50)	1980	49
Haemorrhoids (K64)	655	46
Other anaemias (D64)	1274	45
Anal and rectal abscess (K61)	1214	44
Diverticular disease (K57)	3234	44
Intervertebral disc disorders (M51)	2156	44
Mononucleosis (B27)	517	42
Phlebitis and thrombophlebitis (I80)	1428	42
Localized swelling, head (R22)	523	41
Venous embolism and thrombosis (I82)	548	39
Excessive vomiting in pregnancy (O21)	1205	39
Gout (M10)	659	38
Malaise and fatigue (R53)	516	38
Other spondylopathies (M48)	735	37
Ulcerative colitis (K51)	969	37
Disturbances of skin sensation (R20)	745	36
Facial nerve disorders (G51)	516	36
Cutaneous abscess (L02)	1509	35
Heart failure (I50)	7392	35
Osteomyelitis (M86)	526	34
b) OOH doctor contact before admission (*N* = 152,800)		
	Admissions with the discharge diagnose	OOH contact before admission
Diagnosis	N	%
Acute appendicitis (K35)	5642	52
Foreign body in alimentary tract (T18)	690	52
Effects of other external causes (T75)	732	51
Mental/alcohol disorders (F10)	5779	49
Mental/psychoactive subst. Disorders (F19)	1717	49
Acute tonsillitis (J03)	1130	48
Acute pancreatitis (K85)	1995	46
Abdominal and pelvic pain (R10)	15,518	46
Haemorrhage, airways (R04)	1129	46
Mental/opioids disorders (F11)	757	46
Viral intestinal infections (A08)	1433	46
Adverse effects (T78)	1419	45
Viral infection of unspecified site (B34)	1065	44
Cholelithiasis (K80)	5002	44
Gatroenteritis and colitis (A09)	3225	44
Asthma (J45)	2100	43
Calculus of kidney (N20)	3324	43
Disorders of vestibular function (H81)	2017	43
Paralytic ileus/ intestinal obstruction (K56)	3356	42
Dorsalgia (M54)	3648	42
c) Direct admissions except the ICD-10 diagnosis groups *pregnancy, childbirth and* the puerperium (0XX), and *factors influencing health status and contact with health services (ZXX)* (*N* = 211,551)		
	Admissions with the discharge diagnose	Direct admission
Diagnosis	N	%
Agranulocytosis (D70)	749	72
Hydrocephalus (G91)	587	68
Malignant neoplasms (C)	24,190	63
Cardiac arrest (I46)	539	63
Orthopaedic complications (T84)	2001	62
Pneumonitis due to food and vomit (J69)	836	59
Intracerebral haemorrhage (I61)	1421	58
Fracture of femur (S72)	9958	58
Superficial injury of thorax (S20)	522	58
Mental/sedatives dissorders (F13)	658	58
Epilepsy (G40)	3874	57
Multiple sclerosis (G35)	969	55
Open wound of head (S01)	849	55
Respiratory failure, unspecified (J96)	2388	55
Complications of procedures ICA (T81)	5820	54
Chronic ischaemic heart disease (I25)	2954	54
Chronic kidney disease (N18)	2080	53
Sequelae of cerebrovascular disease (I69)	828	53
Parkinson’s disease (G20)	661	53
Aortic aneurysm and dissection (I71)	982	53

## Discussion

### Main results

We found that 25% of emergency-admitted patients to somatic hospitals in Norway in 2014 were referred by a GP and 31% by an OOH doctor. The largest group of patients were admitted without a registered contact prior to admission (direct admission, 43%). While referrals from GPs were most frequent during office hours, OOH doctors referred patients mainly during evenings, nights and weekends. Direct admissions had the same diurnal pattern as the total emergency admissions, more admissions in daytime and less during the night. Fewer patients living in the most central region were referred by GPs than in less central regions (16% versus 24–31%). More patients were directly admitted (52%) in the most central areas.

When analysing the prehospital paths for different discharge diagnoses, we found considerable variation. It is likely that the explanation for this lies in the nature of the clinical presentation and urgency of the medical conditions, in addition to health service factors. Similar to the findings of Vest-Hansen et al. in Denmark, this study showed that pneumonia was the most common admitted emergency medical condition [25].

### Strengths and limitations

Our study includes all residents of Norway, and all their GP- and OOH contacts, and all emergency admissions to somatic hospitals in 2014. Hence, there is no selection bias. The registries used are based on data delivered with the purpose of managing funding of primary- and specialist healthcare and are therefore probably complete. This means that the material is fully representative for Norway.

There is no information of referring services in the NPR, and we therefore had to make an algorithm for this purpose. The algorithm linked 57% of all emergency admissions to a referring service. Some of the prehospital contacts categorized as referring contacts might be random contacts with no connection to the admission. Nevertheless, we found a clear accumulation of contacts within the 24 h before admission, reducing the likeliness for high incidence of random linkage. Some prehospital contacts with GP or OOH services may not provide sufficient help, leading patients to contact EMCC, which might result in a direct admission by ambulance services. However, only for the most urgent cases would this comply with the national admission routines.

We used the discharge diagnosis to describe the medical condition for each admission. This does not give accurate information about the clinical presentation at the time of admission, which is the basis for deciding the prehospital path. Using the referral diagnosis from the gatekeeping GP and OOH doctor could put extra information on this, but the 43% direct admission would not have such a referral diagnosis. Reasons for encountering GPs or OOH services are not generally available in Norway, and it is thus not possible to link e.g. abdominal pain, fever, etc. to the referral situation.

### Gatekeeping

Generally, a gatekeeping system gives power to primary care doctors (GPs and OOH doctors) to decide whether a patient needs specialty care, hospital care, or a diagnostic test, and patients not have access to specialist or hospital care without a prior examination and a referral [26]. Gatekeeping is associated with lower utilization of health services and has been suggested to reduce hospitalizations [15]. In a healthcare system facing capacity problems, this is a preferred development. Recently there has been debate on the value of gatekeeping related to GPs’ workload and patient choice [14]. Although Norway has a gatekeeper-based healthcare system, we found that only 56% of the emergency-admitted patients came through the primary healthcare gatekeeping system. This is in line with the findings of Grondal et al. from a smaller study at a medical department in Norway, where GPs and OOH doctors referred 26 and 31%, respectively [17]. A reasonable level of gatekeeping for emergency admissions is not possible to determine. However, the variation by centrality could indicate that primary care doctor gatekeeping can be obtained for two thirds of emergency admissions. This could reduce the workload and expenses in hospital care [14].

The diagnoses where the GP played a major role as gatekeeper in our material were anaemias, of which 45–49% of the patients were referred by GP, infections (34–44%) and worsening of chronic disease (34–38%). These diagnoses seem to be less urgent, and might be identified at a regular control consultation, or an extra emergency contact at the GP office. This resembles the picture from Denmark where anaemia, diabetes, atrial fibrillation and heart failure show a reduction in admission rate from office-hours when GPs work, to evening, night and weekend [25]. Skarshaug et al. found a similar pattern in another Norwegian study, showing that 74% of the patients admitted with heart failure had a GP contact within the previous month [27].

The OOH doctor more often was referring patients with conditions where medical investigation and treatment is more urgent, like abdominal pain (42–52%) and mental illness/substance abuse and intoxication (46–49%).

### Direct admissions

The direct admissions are the most frequent prehospital path in our material, and may represent admissions from nursing homes, admissions initiated by hospital doctors following up the patients in specialist healthcare, or directly admitted by ambulance services. As expected, direct admissions are more frequent for highly urgent conditions such as cardiac arrest (63%) and intracerebral haemorrhage (58%) suggesting direct admissions by ambulance service. Our study also shows that 37 and 42% of these cases, respectively, do have a GP or OOH contact before admission. According to national guidelines, cerebral infarction should be managed by direct prehospital path [28]. However, 23% were referred by GPs and 25% by OOH doctors. A study from The Netherlands found that as many as 49% of patients with acute stroke had a GP contact before admission [29]. Probably, some of these patients contact their GP or other primary care providers instead of EMCC in emergencies. The clinical presentation of such urgent conditions is not always the classic acute pattern, similar to stroke and acute coronary syndrome [29, 30].

On the other hand, we know that the OOH doctors and GPs are highly involved in acute cases. In 2014, 65% of the Norwegian OOH services reported that the doctors participate in emergency callouts always or often, when alarmed [22]. One earlier study showed that GPs or OOH doctors participated in 42% of alerted emergency cases [31, 32]. In 2015, the new emergency medicine regulation in Norway stated that the OOH doctors are obliged to be contacted in the emergency communication system and to participate in emergency callouts, when needed [21].

Some medical conditions are followed up in specialist care at hospitals. It is likely that worsening or complications may be discovered at specialist care consultations, or by the patient’s direct contact to the hospital. This might contribute to the high proportion of direct admissions for malignant neoplasms (63%) and orthopaedic complications (62%). Grondal et al. found that 18% of all admissions to a medical department were from outpatient clinics and open return agreements [17]. It is likely that admissions from outpatient clinics at the hospital are often converted for administrative reasons directly from an outpatient contact to an emergency admission without registering the outpatient clinic contact. Also, some of the patients with a discharge diagnosis of malignant disease might have been admitted because of acute symptoms, and then diagnosed with cancer during the hospital stay. Again, these patients would, according to national procedures, usually have been guided by the EMCC or OOH services to a primary care doctor to get a medical examination and referral.

Hip fracture (S72) had a high proportion of direct admissions (58%), illustrating a condition where GP or OOH consultation often is not necessary in order to reveal the need for hospital care. This supports the finding of Skarshaug et al. where 50% of patients urgently admitted to hospital with hip fracture had no GP or OOH contact the month prior to emergency admission [27].

Referrals from nursing home doctors are not specified in our material but included in the direct admissions. We found the same proportion of direct admissions for patients between the age of 80–89 years as for the total population (43%), and only slightly increased direct admissions (47%) for patients 90 years and older. This indicates that admissions from nursing home doctors do not significantly affect the proportion of direct admissions.

### Time of the day

The gatekeeping function was delivered by the GPs and OOHs doctor according to activity in the services, GP in the opening hours, and OOH doctors the rest of the week. The gatekeeper activity is slightly higher than direct admissions throughout the day, with a period in the morning, both on weekdays and weekends, where the direct admissions are more frequent than GP and OOH referrals. This might be because some emergencies are discovered in the morning when the patient and the relatives wake up, or by that the OOH and GP services have less capacity in the transition time between nightshift and daytime work.

### Centrality

GPs and OOH doctors participate less in the emergency callouts in the most central regions in Norway [31, 32]. This may explain the low gatekeeper activity of GPs in the central area, but we did not find the same effect for OOH doctors. Thus, hyper-acute cases with callouts represent relatively few admissions, and therefore the effect of this is relatively sparse. The GPs’ low share of referrals to hospitals may rather be due to GPs in most central regions being less accessible for urgent consultations than their more rural colleagues, but this is not possible to investigate in the present study. Unlike Bankart et al. we found higher rates of emergency admissions in rural areas [7].

### Interpretations

Based on our findings, Norwegian GPs and OOH doctors are gatekeepers in fewer emergency admissions to somatic hospitals than expected, when taking into account the rather strict gatekeeping system that is principally in place. The direct prehospital path representing admissions from ambulance services, referrals from nursing home doctors, and admissions initiated by hospital doctors, represent a larger part of the emergency admissions. This should be taken into account when planning health care services, including strategies in order to reduce hospital overload. On the other hand, there are many clinical conditions where both GPs’ and OOH doctors’ gatekeeping role are considerable.

## Conclusions

GPs or OOH doctors referred many emergencies to somatic hospitals, and for some clinical conditions GPs’ and OOH doctors’ gatekeeping role was considerable. GP referrals were less frequent in the most central areas. A significant number of the emergency admissions had no GP or OOH doctor contact before admission. These direct admissions were more frequent in central areas, for highly urgent conditions and conditions likely to be followed up in specialist care at hospital. The proportion of direct admissions reduces the impact of the GPs’ and OOH doctors’ gatekeeper roles on emergency admissions, even in a strict gatekeeping system.

## Data Availability

The data used in this study are available from The Norwegian Directorate of Health (www.helsedirektoratet.no/) and Statistics Norway (www.ssb.no), but restrictions apply to the availability of these data, which were used under licence for the current study, and so are not publicly available. However, data are available from the authors upon reasonable request and with included permission from The Norwegian Directorate of Health, Statistics Norway, the Regional Ethical Committee, and Norwegian Data Protection Authority.

## Abbreviations

EMCC: Emergency medical communication centre
GP: General practitioner
HELFO: Norwegian Health Economics Administration
ICD-10: The International Statistical Classification of Diseases and Related Health Problems version 10
KUHR: Control and Payment of Reimbursement to Health Service Providers database
NPR: Norwegian Patient Registry
OOH: Out-of-hours
PSPC: Private specialist with public contract
RGP: Regular general practitioner
SSB: Statistics Norway
