# Correction to: General practitioners’ and out-of-hours doctors’ role as gatekeeper in emergency admissions to somatic hospitals in Norway: registry-based observational study

**DOI:** 10.1186/s12913-020-05590-y

**Published:** 2020-09-16

**Authors:** Jesper Blinkenberg, Sahar Pahlavanyali, Øystein Hetlevik, Hogne Sandvik, Steinar Hunskaar

**Affiliations:** 1 National Centre for Emergency Primary Health Care, NORCE Norwegian Research Centre, Kalfarveien 31, 5018 Bergen, Norway; 2 Department of Global Public Health and Primary Care, University of Bergen, Kalfarveien 31, 5018 Bergen, Norway

**Correction to: BMC Health Serv Res 19, 568 (2019)**

**https://doi.org/10.1186/s12913-019-4419-0**

Following publication of the original article [1], the authors would like to correct several numbers in the following paragraphs. In addition, Fig. 1, Fig. 2, Fig. 3, Table 1, Table 2, Table 3, and Table 4 need to be corrected as well.

The numbers need to be corrected for two reasons, both technical and originating from the preparation of the research data files. First, the authors discovered that the material missed data for 8% of primary care contacts due to a data transfer error between the university and the main public data registry. Second, technical personnel in our data centre had made an error in the algorithm when linking datasets, leading to less prehospital contacts linked to emergency hospital admissions. As a result, most numbers in the Results, Tables and Figures were affected. Most of the changes were, however, of insignificant magnitude, and the errors did not affect the main conclusions of the article.

The updated paragraphs are given below, and include the whole results and discussion section, as well as all tables and figures.

**1. Results in the Abstract:**

**Results:** In 2014 there were 497,845 emergency admissions to somatic hospitals in Norway after excluding birth related conditions. Referrals by OOH doctors were most frequent (36%), 35% were direct admissions, 28% were referred by GPs, whereas only 2% were referred from outpatient clinics or private specialists with public contract. Direct admissions were more common in central areas (45%), here GPs’ referrals constituted only 18%. The prehospital paths varied with the hospital discharge diagnosis. For anaemias, 52–56% were referred by GPs, for acute appendicitis and mental/alcohol related disorders 57% and 56% were referred by OOH doctors, respectively. For malignant neoplasms 56% and cardiac arrest 57% were direct admissions

**2. Results in main text:**

**Results**

There were 551,753 emergency hospital admissions to somatic hospitals in Norway in 2014, according to our case definition. One in ten admissions were birth related, hence not supposed to have visited a primary healthcare doctor before admission (Fig. 1). After excluding the birth-related admissions from the material, the distribution of the remaining 497,845 somatic emergency hospital admissions by referring agents is shown in Fig. 1. Referrals by OOH doctors were most frequent (36%), 35% were direct admissions, 28% were referred by GPs, whereas only 2% were referred from outpatient’s clinics or PSPCs.

**Day and time of admission**

Large differences in prehospital paths were found for weekdays vs. weekends, and by day and night hours (Fig. 2). On weekdays, most patients were admitted during the daytime, 53% from 8 am to 4 pm. GP contacts were the main prehospital path in this period, with a little dip representing lunch hour. No patients were admitted from GPs during weekends. Patients referred from the OOH services were the largest group during evenings and nights on weekdays, and all weekends. Direct admissions were high during morning hours and midday, both weekdays and weekends.

**Centrality patterns**

Table 1 and Table 2 show emergency admissions by centrality group, referring agent, and per 1000 inhabitants. The mean number of emergency admissions per 1000 inhabitants per year was 97, highest in the least central group (115), and lowest in the most central group (87). For direct admissions, we found an increasing proportion by increasing centrality, so in the most central (urban) areas almost half of the admissions to somatic hospitals in 2014 were direct admissions. For the two least central areas, with 12% of the population and 14% of the admissions, only 28% of the admissions were direct.

There was an increasing proportion of referrals from GPs by decreasing centrality, as referrals from GPs constituted only 18% in the most central group and 34% of the admissions in the two least central groups of municipalities. The proportion of patients referred from OOH doctors was relatively stable by centrality group, varying from 32 to 37% in the various centrality groups. Outpatient clinics and PSPCs referred few patients, and had low shares in all centrality groups, but reached 4% in the most central group. Hospitals in the most central regions had up to 57% direct admissions, whereas the most rural had only 22% (data not shown).

**Diagnoses**

Among all the emergency admissions, injuries were the most frequent discharge diagnosis group, followed by diseases in the circulatory system, symptoms and findings not elsewhere classified, and diseases in the respiratory system (Fig. 3).

Table 3 shows the 20 most common diagnoses by the four prehospital paths, these diagnoses constituted 35% of all admissions. Pneumonia (J15, J18) was the most common diagnosis, followed by pain in throat and chest (R07), abdominal and pelvic pain (R10), atrial arrhythmias (I48), and acute myocardial infarction (I21). Several kinds of injuries were also in the top 20, together with major chronic diseases such as chronic obstructive pulmonary disease (COPD) and heart failure.

Prehospital paths differed considerably between different discharge diagnoses (Table 4). The GPs (28% of all emergency admissions) had a much higher share of, e.g. anaemias and other conditions of the blood, sciatica, heart failure, and various local subacute diseases like haemorrhoids, diverticulitis, and deep venous thrombosis. OOH doctors (36% of all admissions) had a high share of referrals for various acute conditions, like appendicitis, foreign body in alimentary tract, mental and alcohol related disorders, abdominal pain and other acute gastro-intestinal conditions, asthma, and nephrolithiasis. The direct prehospital path (35% of all admissions) was most common for the diagnosis of agranulocytosis, hydrocephalus and cardiac arrest, but all with relatively small absolute numbers. The top seven diagnoses with direct admissions had a percentage above 50, revealing a list of conditions being extensively removed from undergoing a gatekeeper process. Admissions for malignant neoplasms was by far the largest group(C) (56%, *N* = 24,190), followed by fractures and other orthopedic conditions, epilepsy, and chronic diseases of the lungs, kidneys and heart. Major and common emergencies, such as stroke (42%), acute myocardial infarction (42%) and pneumonia (29%) did not reach the top 20 list of direct admissions but had high absolute numbers.

**3. Discussion in main text**

**Main results**

We found that 28% of emergency-admitted patients to somatic hospitals in Norway in 2014 were referred by a GP and 36% by an OOH doctor. The second largest group of patients were admitted without a registered contact prior to admission (direct admission, 35%). While referrals from GPs were most frequent during office hours, OOH doctors referred patients mainly during evenings, nights and weekends. Direct admissions had the same diurnal pattern as the total emergency admissions, more admissions in daytime and less during the night. Fewer patients living in the most central region were referred by GPs than in less central regions (18% versus 27–34%). More patients were directly admitted (45%) in the most central areas.

When analysing the prehospital paths for different discharge diagnoses, we found considerable variation. It is likely that the explanation for this lies in the nature of the clinical presentation and urgency of the medical conditions, in addition to health service factors. Similar to the findings of Vest-Hansen et al. in Denmark, this study showed that pneumonia was the most common admitted emergency medical condition (25).

**Strengths and limitations**

Our study includes all residents of Norway, and all their GP- and OOH contacts, and all emergency admissions to somatic hospitals in 2014. Hence, there is no selection bias. The registries used are based on data delivered with the purpose of managing funding of primary- and specialist healthcare and are therefore probably complete. This means that the material is fully representative for Norway.

There is no information of referring services in the NPR, and we therefore had to make an algorithm for this purpose. The algorithm linked 65% of all emergency admissions to a referring service. Some of the prehospital contacts categorized as referring contacts might be random contacts with no connection to the admission. Nevertheless, we found a clear accumulation of contacts within the 24 h before admission, reducing the likeliness for high incidence of random linkage. Some prehospital contacts with GP or OOH services may not provide sufficient help, leading patients to contact EMCC, which might result in a direct admission by ambulance services. However, only for the most urgent cases would this comply with the national admission routines.

We used the discharge diagnosis to describe the medical condition for each admission. This does not give accurate information about the clinical presentation at the time of admission, which is the basis for deciding the prehospital path. Using the referral diagnosis from the gatekeeping GP and OOH doctor could put extra information on this, but the 35% direct admission would not have such a referral diagnosis. Reasons for encountering GPs or OOH services are not generally available in Norway, and it is thus not possible to link e.g. abdominal pain, fever, etc. to the referral situation.

**Gatekeeping**

Generally, a gatekeeping system gives power to primary care doctors (GPs and OOH doctors) to decide whether a patient needs specialty care, hospital care, or a diagnostic test, and patients not have access to specialist or hospital care without a prior examination and a referral (26). Gatekeeping is associated with lower utilization of health services and has been suggested to reduce hospitalizations (15). In a healthcare system facing capacity problems, this is a preferred development. Recently there has been debate on the value of gatekeeping related to GPs’ workload and patient choice (14). Although Norway has a gatekeeper-based healthcare system, we found that only 65% of the emergency-admitted patients came through the primary healthcare gatekeeping system. This is in line with the findings of Grondal et al. from a smaller study at a medical department in Norway, where GPs and OOH doctors referred 26 and 31%, respectively (17). A reasonable level of gatekeeping for emergency admissions is not possible to determine. However, the variation by centrality could indicate that primary care doctor gatekeeping can be obtained for two thirds of emergency admissions. This could reduce the workload and expenses in hospital care (14).

The diagnoses where the GP played a major role as gatekeeper in our material were anaemias, of which 52–56% of the patients were referred by GP, infections (39–47%) and worsening of chronic disease (39–44%). These diagnoses seem to be less urgent, and might be identified at a regular control consultation, or an extra emergency contact at the GP office. This resembles the picture from Denmark where anaemia, diabetes, atrial fibrillation and heart failure show a reduction in admission rate from office-hours when GPs work, to evening, night and weekend (25). Skarshaug et al. found a similar pattern in another Norwegian study, showing that 74% of the patients admitted with heart failure had a GP contact within the previous month (27).

The OOH doctor more often was referring patients with conditions where medical investigation and treatment is more urgent, like abdominal pain (47–57%) and mental illness/substance abuse and intoxication (54–56%).

**Direct admissions**

The direct admissions are the second most frequent prehospital path in our material, and may represent admissions from nursing homes, admissions initiated by hospital doctors following up the patients in specialist healthcare, or directly admitted by ambulance services. As expected, direct admissions are more frequent for highly urgent conditions such as cardiac arrest (57%) and intracerebral haemorrhage (51%) suggesting direct admissions by ambulance service. Our study also shows that 43 and 49% of these cases, respectively, do have a GP or OOH contact before admission. According to national guidelines, cerebral infarction should be managed by direct prehospital path (28). However, 27% were referred by GPs and 31% by OOH doctors. A study from The Netherlands found that as many as 49% of patients with acute stroke had a GP contact before admission (29). Probably, some of these patients contact their GP or other primary care providers instead of EMCC in emergencies. The clinical presentation of such urgent conditions is not always the classic acute pattern, similar to stroke and acute coronary syndrome (29, 30).

On the other hand, we know that the OOH doctors and GPs are highly involved in acute cases. In 2014, 65% of the Norwegian OOH services reported that the doctors participate in emergency callouts always or often, when alarmed (22). One earlier study showed that GPs or OOH doctors participated in 42% of alerted emergency cases (31, 32). In 2015, the new emergency medicine regulation in Norway stated that the OOH doctors are obliged to be contacted in the emergency communication system and to participate in emergency callouts, when needed (21).

Some medical conditions are followed up in specialist care at hospitals. It is likely that worsening or complications may be discovered at specialist care consultations, or by the patient’s direct contact to the hospital. This might contribute to the high proportion of direct admissions for malignant neoplasms (56%) and orthopaedic complications (54%). Grondal et al. found that 18% of all admissions to a medical department were from outpatient clinics and open return agreements (17). It is likely that admissions from outpatient clinics at the hospital are often converted for administrative reasons directly from an outpatient contact to an emergency admission without registering the outpatient clinic contact. Also, some of the patients with a discharge diagnosis of malignant disease might have been admitted because of acute symptoms, and then diagnosed with cancer during the hospital stay. Again, these patients would, according to national procedures, usually have been guided by the EMCC or OOH services to a primary care doctor to get a medical examination and referral.

Hip fracture (S72) had a high proportion of direct admissions (47%), illustrating a condition where GP or OOH consultation often is not necessary in order to reveal the need for hospital care. This supports the finding of Skarshaug et al. where 50% of patients urgently admitted to hospital with hip fracture had no GP or OOH contact the month prior to emergency admission (27).

Referrals from nursing home doctors are not specified in our material but included in the direct admissions. We found the same proportion of direct admissions for elderly patients as for the total population, 80–89 years 33, and 34% for patients 90 years and older. This indicates that admissions from nursing home doctors do not significantly affect the proportion of direct admissions.

**Time of the day**

The gatekeeping function was delivered by the GPs and OOHs doctor according to activity in the services, GP in the opening hours, and OOH doctors the rest of the week. The gatekeeper activity is higher than direct admissions throughout the day, with a period in the morning, both on weekdays and weekends, where the direct admissions are as frequent as GP and OOH referrals. This might be because some emergencies are discovered in the morning when the patient and the relatives wake up, or by that the OOH and GP services have less capacity in the transition time between nightshift and daytime work.

**Centrality**

GPs and OOH doctors participate less in the emergency callouts in the most central regions in Norway (31, 32). This may explain the low gatekeeper activity of GPs in the central area, but we did not find the same effect for OOH doctors. Thus, hyper-acute cases with callouts represent relatively few admissions, and therefore the effect of this is relatively sparse. The GPs’ low share of referrals to hospitals may rather be due to GPs in most central regions being less accessible for urgent consultations than their more rural colleagues, but this is not possible to investigate in the present study. Unlike Bankart et al. we found higher rates of emergency admissions in rural areas (7).

**Interpretations**

Based on our findings, Norwegian GPs and OOH doctors are gatekeepers in fewer emergency admissions to somatic hospitals than expected, when taking into account the rather strict gatekeeping system that is principally in place. The direct prehospital path representing admissions from ambulance services, referrals from nursing home doctors, and admissions initiated by hospital doctors, represent a large part of the emergency admissions. This should be taken into account when planning health care services, including strategies in order to reduce hospital overload. On the other hand, there are many clinical conditions where both GPs’ and OOH doctors’ gatekeeping role are considerable.

**Fig. 1:**
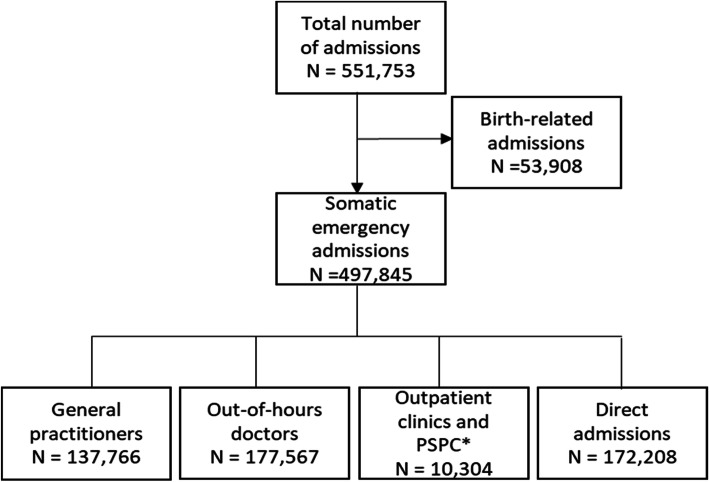
Prehospital pathways for all the emergency admissions to somatic hospitals in Norway in 2014 *Private specialist with public contract

**Fig. 2:**
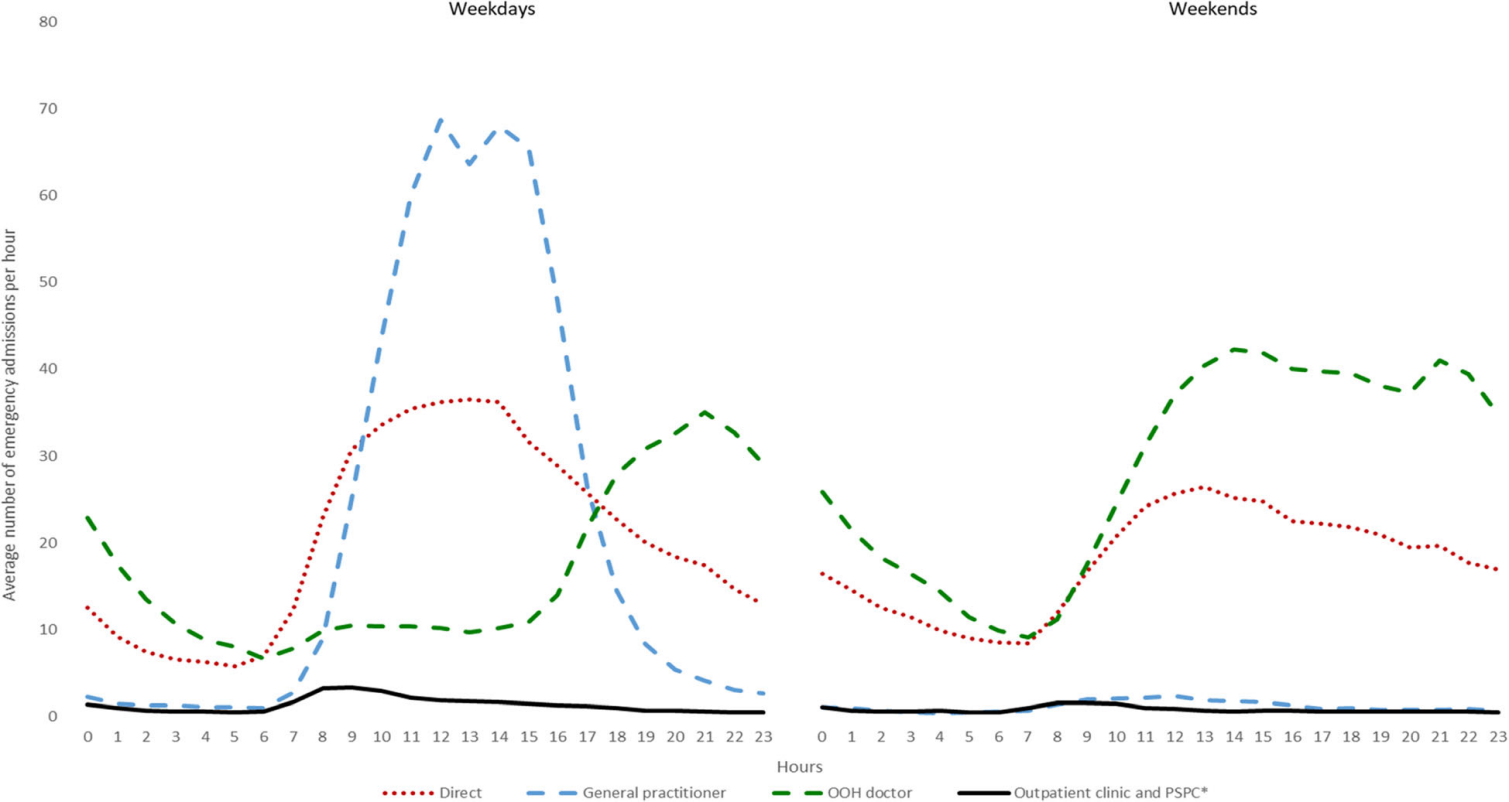
All emergency admissions to somatic hospitals in 2014 in Norway, sorted by prehospital pathways and time of the day during weekdays and weekends

**Fig. 3:**
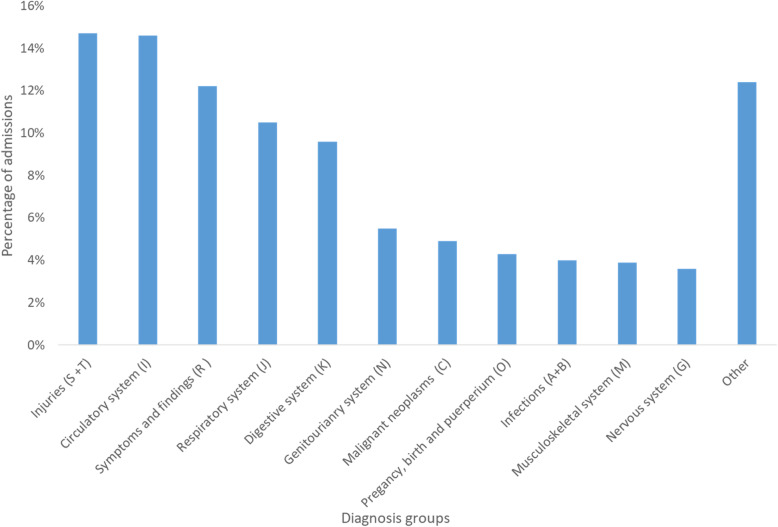
Distribution of admissions by diagnosis groups for the discharge diagnosis (ICD-10) after emergency admissions to somatic hospitals (except normal birth and related conditions) in Norway 2014 (*N* = 497,845)

**Table 1:** Frequency of all emergency admissions to somatic hospitals in Norway 2014 by patient residence centrality

Centrality	All admissions		Population	
	N	%	N	Admissions per 1000
1 (most central)	88,086	18	1,011,602	87
2	122,043	25	1,199,290	102
3	124,055	25	1,357,164	91
4	94,456	19	906,580	104
5	48,982	10	459,368	107
6 (least central)	20,107	4	175,052	115
Sum	497,729^a^	100	5,109,056	97

^a^116 cases missing the centrality variable

**Table 2:** Variation in prehospital paths by patient residence centrality for all emergency admissions to somatic hospitals in Norway 2014 (*N* = 497,729^a^)

	General practitioner		Out-of-hours doctor		Outpatient clinic or PSPC^**b**^		Direct admission	
Centrality	N	%	N	%	N	%	N	%
1 (most central)	15,820	18	28,596	32	3828	4	39,842	45
2	32,363	27	45,181	37	2098	2	42,401	35
3	36,372	29	43,537	35	2045	2	42,101	34
4	29,651	31	34,676	37	1537	2	28,592	30
5	16,708	34	18,104	37	606	1	13,564	28
6 (least central)	6845	34	7424	37	189	1	5649	28

^a^116 cases missing the centrality variable

^b^Private specialist with public contract

**Table 3:** Distribution of prehospital pathways for all admissions (except birth related conditions), and by discharge diagnosis (ICD-10 codes) for the 20 most common diagnosis after somatic hospital stays in Norway 2014

	General practitioner		Out-of-hours doctor		Outpatient clinic or PSPC^**a**^		Direct admission		Sum	
	N	%	N	%	N	%	N	%	N	%
All admissions	137,766	28	177,567	36	10,304	2	172,208	35	497,845	100
**Diagnosis (ICD-10)**										
Pneumonia (J15 + J18)	6499	32	7918	39	109	1	5962	29	20,488	100
Pain in throat and chest (R07)	4710	29	7613	47	74	0	3923	24	16,320	100
Abdominal and pelvic pain (R10)	4930	32	7874	51	81	1	2633	17	15,518	100
Atrial fibrillation and flutter (I48)	4423	37	3885	33	170	1	3395	29	11,873	100
Acute myocardial infarction (I21)	2699	24	3814	34	92	1	4705	42	11,310	100
Fracture of femur (S72)	1634	16	3417	34	192	2	4715	47	9958	100
Chronic obstructive pumonary disease (J44)	2743	30	3461	38	45	0	2754	31	9003	100
Intracranial injury (S06)	1178	14	3734	45	316	4	3021	37	8249	100
Other dissorders of urinary system (N39)	2233	30	3158	42	49	1	2058	27	7498	100
Cerebral infarction (I63)	1973	27	2313	31	45	1	3078	42	7409	100
Heart failure (I50)	2935	40	2191	30	72	1	2194	30	7392	100
Angina pectoris (I20)	2107	31	2253	33	113	2	2277	34	6750	100
Complications of procedures (T81)	1257	22	1581	27	174	3	2808	48	5820	100
Alcohol related disorders (F10)	641	11	3262	56	24	0	1852	32	5779	100
Acute appendicitis (K35)	1827	32	3233	57	9	0	573	10	5642	100
Syncope and collapse (R55)	1305	25	2240	42	45	1	1704	32	5294	100
Choleolithiasis (K80)	1549	31	2488	50	22	0	943	19	5002	100
Medical observation (Z03)	1567	32	1735	35	52	1	1560	32	4914	100
Fracture of forearm (S52)	698	15	1992	42	317	7	1770	37	4777	100
Fracture of lower leg, including ancle (S82)	630	13	1858	40	211	5	1983	42	4682	100
Sum	47,538		70,020		2212		53,908		173,678	35 (of all)

^a^Private specialist with public contract

**Table 4:** Emergency admissions by discharge ICD-10 diagnosis where contact with a) GP or b) out-of-hour (OOH) doctor, or c) direct admission is the dominating prehospital pathway

**a) GP contact before admission (*N*** **= 137,766)**		
	**Admissions with the discharge diagnose**	**GP contact before admission**
**Diagnosis**	**N**	**%**
Iron deficiency anaemia (D50)	1980	56
Other anaemias (D64)	1274	52
Haemorrhoids (K64)	655	48
Diverticular disease (K57)	3234	48
Abscess of anal and rectal regions (K61)	1214	47
Intervertebral disc disorders (M51)	2180	47
Localized swelling, head (R22)	523	46
Phlebitis and thrombophlebitis (I80)	1428	46
Gout (M10)	659	44
Mononucleosis (B27)	517	43
Other spondylopathies (M48)	735	43
Venous embolism and thrombosis (I82)	548	43
Excessive vomiting in pregnancy (O21)	1205	42
Malaise and fatigue (R53)	516	41
Ulcerative colitis (K51)	969	40
Heart failure (I50)	7392	40
Atherosclerosis (I70)	1097	39
Disturbances of skin sensation (R20)	745	39
Facial nerve disorders (G51)	516	39
Osteomyelitis (M86)	526	39
**b) OOH doctor contact before admission (*N*** **= 177,567)**		
	**Admissions with the discharge diagnose**	**OOH contact before admission**
**Diagnosis**	**N**	**%**
Foreign body in alimentary tract (T18)	690	60
Mental/psychoactive subst. disorders (F19)	1717	58
Effects of other external causes (T75)	732	58
Acute appendicitis (K35)	5642	57
Mental/alcohol disorders (F10)	5779	56
Mental/opioids disorders (F11)	757	54
Acute tonsillitis (J03)	1130	53
Haemorrhage, airways (R04)	1129	53
Acute pancreatitis (K85)	1995	52
Viral intestinal infections (A08)	1433	51
Abdominal and pelvic pain (R10)	15,518	51
Cholelithiasis (K80)	5002	50
Adverse effects (T78)	1419	50
Viral infection of unspecified site (B34)	1065	49
Gastroenteritis and colitis (A09)	3225	49
Paralytic ileus / intestinal obstruction (K56)	3356	48
Disorders of vestibular function (H81)	2017	48
Asthma (J45)	2100	48
Dorsalgia (M54)	3648	47
Calculus of kidney (N20)	3324	47
**c) Direct admissions except the ICD-10 diagnosis groups** ***pregnancy, childbirth and*** **the puerperium (0XX), and** ***factors influencing health status and contact with health services (ZXX)*** **(*N*** **= 172,208)**		
	**Admissions with the discharge diagnose**	**Direct admission**
**Diagnosis**	**N**	**%**
Agranulocytosis (D70)	749	66
Hydrocephalus (G91)	587	64
Cardiac arrest (I46)	539	57
Malignant neoplasms (C)	24,190	56
Orthopaedic complications (T84)	2001	54
Superficial injury of thorax (S20)	522	53
Intracerebral haemorrhage (I61)	1421	51
Mental/sedatives dissorders (F13)	658	49
Open wound of head (S01)	849	49
Multiple sclerosis (G35)	969	49
Complications of procedures ICA (T81)	5820	48
Epilepsy (G40)	3874	48
Fracture of femur (S72)	9958	47
Chronic ischaemic heart disease (I25)	2954	47
Aortic aneurysm and dissection (I71)	982	46
Fracture of skull and facial bones (S02)	1132	45
Pleural effusion, not elsewhere classified (J90)	915	45
Nonrheumatic aortic valve disorders (I35)	1280	44
Convulsions, not elsewhere classified (R56)	1838	44
Pneumonitis due to food and vomit (J69)	836	44
